# Workplace violence in a tertiary care Israeli hospital - a systematic analysis of the types of violence, the perpetrators and hospital departments

**DOI:** 10.1186/s13584-017-0168-x

**Published:** 2017-08-23

**Authors:** Sigal Shafran-Tikva, Revital Zelker, Zvi Stern, David Chinitz

**Affiliations:** 1 Hadassah University Medical Center, P.O.B 12109, Kiryat Hadassah, 12000 Jerusalem, Israel; 2 National Insurance Institute of Israel, Jerusalem, Israel; 3 School of Public Health, Hebrew University-Hadassah, Jerusalem, Israel

**Keywords:** Violence, Nurses, Physicians, General hospital

## Abstract

**Background:**

Worldwide, there is a widespread and disturbing pattern of violence towards healthcare workers. However, violent occurrences in Israeli hospitals have often been unrecognized and underreported. Moreover, most studies have not sufficiently differentiated among the different types of violence.

To examine the different types of violence experienced by nurses and physicians, the types of perpetrators and the specialty fields involved.

**Methods:**

A quantitative questionnaire was used to assess the incidence of a “basket” of violent behaviors, divided into eight types of violent manifestations. The study population consisted of 729 physicians and nurses in a variety of hospital divisions and departments (surgery, oncology, intensive care, ambulatory services including day care, and emergency room) in a large general hospital. Six hundred seventy-eight of them responded to the survey for a response rate of 93%; about two thirds of respondents (446) were nurses and about one third (232) were physicians. The questionnaires were completed during staff meetings and through subsequent follow-up efforts.

**Results:**

In the 6 months preceding the survey, the respondents experienced about 700 incidents of passive aggressive behavior, 680 of verbal violence and 81 of sexual harassment. Types of violence differed between patients and companions; for example, the latter exhibited more verbal, threatening and passive aggressive behaviors. Violence was reported in all departments (ranging from 52–96%), with the departments most exposed to violence being the emergency room and outpatient clinics. Nurses in the emergency room were 5.5 times at a higher risk of being exposed to violence than nurses in the internal medicine department. Nurses were exposed to violence almost twice as much as physicians. There was a positive association between the physician’s rank and his/her exposure to violence. A multiple regression model found that being older reduced the risk of being exposed to violence, for both physicians and nurses.

**Conclusions:**

These findings suggest that uniform definitions of a range of different violent behaviors and assessments of their prevalence are important to creating an improved discourse about hospital violence in both research and operational settings. The study findings could assist policy makers in the Israeli healthcare system in implementing interventions on a national level and can promote leaders’ commitment to violence prevention and management. This is an important contribution, as executive commitment is necessary and critical for the necessary organizational changes to occur.

## Introduction

Aggression and violence in the workplace is an underreported, global problem that has been tolerated and largely ignored [1]. In the United States, for example, violence was found to be a significant contributor to injuries and death on the job [2, 3]. Indications are that rates of workplace violence are increasing, and probably at a higher rate in the health care relative to other settings [4] The damage caused by violence translates into high costs for the organization and physical and mental harm to the victim in the short and long term [5–8]. Violence in the workplace and in hospitals in particular is detrimental not only to the organization but also to the worker. According to the World Health Organization [9], violence against the healthcare staff may also have a negative effect on the quality of care provided to violent patients. A study conducted by Roche [10] (2010) found that violence is unfortunately a major component of the nurse’s work life and that it negatively affects nursing job satisfaction, the climate in the department and outcomes of patient care.

### Definitions of violent behavior and aggression

The research literature offers many definitions for the terms violence and aggression. For the purposes of this paper violence is defined as a socially unacceptable behavior - aggressive and sometimes destructive - of an individual or group. Frustration, hostility and prejudice might serve as catalysts for violent behavior. Aggression can be an innate behavior or a response to frustration leading to self-assertion. Aggressive behavior can be destructive and aggressive or covert hostile behavior [11, 12].

Although the above definition seems clear, there is no consensus in the literature as to which behaviors are defined as violent or aggressive behaviors, mainly with regard to violence in the workplace [13]. Some researchers suggest that aggression in general is one instrument for achieving either instrumental or affective goals, hat sometimes takes the specific form of violence aimed at inflicting physical or psychological arm or at the very least to be insulting and threatening [14].

## Background

The literature reports on diverse types of violence aimed at the medical staff in various countries. The published studies show that patients and those who accompany them demonstrate a variety of types of violence towards nurses, physicians and other healthcare workers. The prevalent types of violence are verbal violence, physical violence, annoyances and sexual harassment [15, 16]. Sometimes violence can extend even to murder as recent evidence from China shows [17]. Hence, violence against the healthcare staff is a wide concept that encompasses different types of behavior displayed in different hospital departments. For this reason, it is extremely important to list the behaviors and to examine their prevalence in each discipline and specialty area.

As mentioned above, various healthcare professionals are subjected to violence to different extents. A considerable part of the studies published to date were conducted by nurses and published in various nursing journals. This suggests that nurses are on the front line more than physicians with regard to exposure to violence. The number of studies focusing on violence in ER suggests that it is more prevalent in the later than in other departments [15, 18]. However, prior to the current study, there was little empirical evidence on these issues.

According to the literature review violence towards healthcare workers crosses borders and cultures, A study that reviewed the prevalence of violence by region found that rates of exposure vary by world region (Anglo, Asia, Europe and Middle East), with the highest rates of physical violence and sexual harassment being in the Anglo region and the highest rates of non-physical violence and bullying in the Middle East [16].

A survey conducted in 65 departments of emergency medicine in the United States found that over a five-year period prior to the study, 3461 physical assaults had occurred in departments of emergency medicine, with guns or knives used in 20% of these incidents [19]. In Iran, the prevalence of violence was examined in five hospitals. The research findings showed that 96% of respondents experienced verbal violence and 29% physical violence over the past 5 years, prior the study [20].

A study conducted in China, examining the extent of violence towards physicians and nurses in 12 hospitals found that some 50% of respondents reported that they had been exposed to some type of violence in the year prior to the study [17]. A study conducted in a large hospital in Britain found that over 68% of the staff reported verbal assaults in the year prior to the study, with nurses (43.4%) reporting more exposure to violence than physicians (13.8%) [19]. In Israel, Landau et al. conducted a study examining all of the emergency rooms in Israel and found that 75% of staff had been exposed to violence in the year prior to the study. The most frequent type of violence displayed towards staff is verbal; 29% of incidents include threats, and physical violence was displayed in 16% of the incidents [21]. Also, Derazon et al. (1999) [22] found that in the ER 74% of the participants in a single hospital in Israel had experienced 5 episodes of violence in the past 2 years. Ninety percent (90%) of the nursing staff experienced some kind of violence (half physical), as did 70% of the physicians and 64% of ER hospital admission personnel. Another study performed in Israeli ER departments found a relationship between violence and stress of offenders and victims. Verbal violence was commonly reported (52%) and physical violence was reported by 10% of respondents during the preceding year [23].

To the best of our knowledge, in Israel, aside from studies limited to emergency rooms, the prevalence of all types of violence in general hospitals has not been measured to date. What is missing is not only an overall assessment of the extent of violence but an unpacking of this phenomenon into different types and their frequencies. While previous work may have differentiated between, for example and as above, violence and aggression, a more penetrating taxonomy is needed if we are to come to grips with the enactment of violence in different micro situations within hospitals. We aimed to fill this gap and provide a deeper understanding of violence in an Israeli hospital. In addition, it is important to compare between the prevalence of types of violence displayed towards physicians and nurses as well as their prevalence in various hospital departments.

Therefore, this study examines the extent of violence along several dimensions:

A. The types of violence - such as, verbal violence, verbal threats, destruction of property, minor physical violence, severe physical violence, use of a weapon or a sharp object and sexual harassment.
B. The perpetrators of violence (patient/accompanying person)
C. The professions (physicians/nurses)
D. The hospital departments - (i.e., fields of specialty).

## Methods

This study was conducted at a university-affiliated medical center with 700 beds, employing some 5000 workers that include approximately 700 physicians and 1000 nurses. The hospital is a tertiary university affiliated medical center that offers advanced services, as well as outpatient clinics in Jerusalem and the vicinity. Approximately 30,000 patients and visitors use the hospital’s services every day.

The hospital is located in Jerusalem and treats patients from a variety of cultures and ethnic groups. The healthcare workers also come from a variety of cultures and customs. Notably, the study was conducted in 2011 and data collection took place in a relatively uneventful period in terms of the Israeli-Palestinian conflict.

The data published in this paper are part of a larger study which included qualitative method. The qualitative component included focus groups and in depth interviews, which informed development of the quantitative questionnaire and provided deeper understanding of the causes and factors that lead to the formation of violence in general hospitals in Israel.

**The research population** included all physicians and nurses from the departments of internal medicine, surgery, oncology, intensive care, ambulatory care and clinics, and the department of emergency medicine, who were asked to complete an anonymous questionnaire.

### Study tool

With the aim of estimating the prevalence and identifying the types of violence demonstrated towards physicians and nurses, the behaviors studied were divided into eight different types.

These types were defined on the basis of a literature review and focus groups with nurses and physicians.

The resulting categories were:

- verbal violence - shouting, insults and curses;
- verbal threats, such as the threat of taking action against the healthcare worker after work;
- passive aggressive behavior - sharp looks, stern facial expressions, muttering;
- minor physical violence - shoving, blocking the way;
- destruction of property in protest - throwing a chair, breaking an instrument, tearing up a medical file;
- severe physical violence - punching, kicking, throwing objects;
- use of a firearm or a knife;
- sexual harassment.

Respondents were asked to refer to the extent of their exposure to violence in the six-month period prior to the study from three aspects: type of violence, frequency of exposure, and the violence perpetrator - patient or person accompanying a patient.

In order to compare the rate of exposure to violence between physicians and nurses, a new summary measure was formed, called “exposure to violence”., defined as being exposed to at least one incident in the past 6 months prior to completing the questionnaire. Also, the questionnaire included demographics such as age, gender, professional seniority, departmental seniority, job percentage and position.

The passive- aggressive behavior category is sometimes excluded from studies of the prevalence of violence, in part because it is based on subjective perceptions. However, we found it to be a major theme in our focus groups, and a potential precursor of other types of violence. The unfolding of violent events in the hospital setting, which is bound up precisely with subjective perceptions, is described in a companion study [24].

#### Content validity

For purposes of validation, the questionnaire was given to three physicians and five nurses from a variety of fields and ethnic origins for their review. An interview was held with each of the reviewers concerning the clarity of the questionnaire and the suitability of its content for the domain that it aimed to examine. After corrections, the questionnaire was given to 11 key personnel from various sectors at the hospital for their comments.

### Questionnaire distribution

Based on an updated list of the physicians and nurses currently working in hospital departments obtained from the department secretariat, the researcher approached potential study participants. Medical as well as nursing managers were contacted in person and upon their advice; the researcher (first author) took part in staff meetings and presented the study’s purpose and procedure. Data collection was carried out in two stages: First, we asked doctors and nurses to fill out the questionnaires in pre-planned departmental meetings. In the second stage we contacted all those who did not participate in a staff meeting (according to the department’s personnel list)and asked them to fill in the questionnaire.

### Description of the statistical methods for data analysis

To describe the association between qualitative variables and other sub-groups (age, professional status, etc.,), a chi-squared test (×2) and fisher’s Exact test were used. An association between two dependent sequential variables was tested using the McNemar test.

For quantitative variables, the relationship between subgroups was made using t-test for two independent groups and the Mann–Whitney test. Multivariate relationships between exposures to violence by patients or those accompanying them and all background variables (age, gender, seniority, professional status, hospital division, etc.) were tested through logistic regression.

## Results

A total of 729 physicians and nurses were approached from the hospital departments, of which 678 participated in the study (a response rate of 93%). A description of the research sample is provided in Table 1. Mean age was 41 year (SD ± 11.2), and 60% were of female. Of the total sample 34% were physicians, of which 16% were seniors, 13% were interns and 3 were department heads. The rest (66%) were nursing staff, 56% staff nurses. Most of the nursing sample worked full time and had professional seniority of 11 + 14 years.

**Table 1:** Distribution of demographic and professional variables

Variable	Description	N (%)	Total (n)
Age (mean yrs.; sd)		41 ± 11	641 (100%)
Gender (n;%)	Male	270 (40%)	677 (100%)
	Female	407 (60%)	
Physicians (n;%)	Department Head	23 (3%)	230 (34%)
	Senior physician	104 (15%)	
	Resident	86 (13%)	
	Intern	19 (3%)	
Nurses (n;%)	Head nurse	33 (5%)	446 (66%)
	Staff nurse	375 (56%)	
% working half-time or more >50% (n;%)		635 (98%)	643 (100%)
Professional seniority (mean yrs.; sd)		11 ± 14	641 (100%)
Departmental seniority (mean yrs.; sd)		8 ± 9	646 (100%)

As seen in Table 4, over 50% of the respondents were employed in the departments of surgery and internal medicine. Approximately 12% were employed in the department of oncology, 11% in intensive care and 9% in ambulatory care services. Of all respondents, only 68 (10.4%) were trained in coping with and preventing violence. Only 125 (19.2%) reported that their department had a procedure for preventing, responding and documenting violent incidents.

### Types and rate of violence

Physicians and nurses were both exposed to violence at a considerable rate (Table 2). One out of three staff members (58%) experienced any type of violence in the last 6 months either by patients or by people accompanying them.

**Table 2:** The extent of exposure to all types of violence during the past 6 months

Type of violence	By patient	By companion
Verbal violence	304 (43.1%)	331 (47%)
Explicit threat	136 (19.3%)	176 (25%)
Passive aggressive	336 (47.7%)	378 (53.6%)
Equipment destruction	67 (9.5%)	65 (9.2%)
Mild violence	42 (6%)	54 (7.7%)
Severe violence	23 (3.3%)	17 (2.4%)
Sharp object	11 (1.6%)	6 (0.9%)
Sexual harassment	47 (6.7%)	34 (4.8%)

For example, during the previous 6 months, there were approximately 700 incidents of passive aggressive behavior, 680 incidents of verbal violence and 81 incidents of sexual harassment. These behaviors were demonstrated towards physicians and nurses both by patients and by people accompanying them. Companions demonstrated more verbal violence, verbal threats, passive aggressive behavior, minor physical violence and sexual harassment compared to patients who demonstrated more violence categorized as destruction of property, severe violence and use of a sharp object. As can be seen from Table 3, respondents often suffered more than one occurrence of violence, with the most frequent “repeat” experiences involving verbal violence, threats and passive aggressive behavior.

**Table 3:** The extent of exposure to all types of violence during the past 6 months, frequencies

Type of violence	By whom	1–2 times	3–5 times	6 times or more
Verbal violence	Patient	204 (29%)	59 (8%)	41 (6%)
	Companion	209 (30%)	83 (12%)	40 (6%)
Explicit threat	Patient	96 (14%)	32 (4%)	8 (1%)
	Companion	121 (17%)	44 (6%)	11 (2%)
Passive aggressive	Patient	208 (30%)	74 (10%)	53 (7%)
	Companion	218 (31%)	100 (14%)	61 (9%)
Equipment destruction	Patient	54 (8%)	11 (2%)	2 (0.3%)
	Companion	56 (8%)	7 (1%)	2 (0.3%)
Mild violence	Patient	37 (5%)	4 (0.6%)	1 (0.1%)
	Companion	51 (7%)	2 (0.3%)	1 (0.1%)
Severe violence	Patient	21 (3%)	1 (0.1%)	1 (0.1%)
	Companion	18 (3%)	0	0
Sharp object	Patient	9 (1%)	2 (1%)	0
	Companion	4 (0.6%)	2 (0.3%)	0
Sexual harassment	Patient	35 (4.8%)	8 (1%)	3 (0.4%)
	Companion	27 (3.8%)	4 (0.6%)	3 (0.4%)

### Nurses and physicians’ exposure to violence by patients and/or companions

A distribution of the prevalence of violent incidents perpetrated by patients and/or companions by profession of the victims, found that nurses are exposed to violence by patients to a significantly greater extent than physicians. In particular, patients and companions assaulted nurses twice as much as physicians. This difference was significant (Chi-square 20.909^b^, df = 6, *P = 0.002*)*.* A similar difference was also found among companions.

Table 4 shows the incidence of violence perpetrated by patients and companions, categorized by types of violent behaviors. Significant differences were found between the experience of physicians and nurses.

**Table 4:** Comparison between physicians and nurses - types of violence

The victim	Explicit threat		Verbal violence	
	Patient *n* = 667	Companion *n* = 670	Patient *n* = 672	Companion *n* = 672
Physicians	27(12%)	34(15%)	78(34%)	91(40%)
Nurses	103(23%)	134(30%)	217(49%)	230(52%)
*P* value	*P* = 0.0003	*P* = 0.00001	*P* = 0.0002	*P* = 0.002

### Correlation between exposure to violence and professional seniority

When comparing among different types of nurses (licensed practical nurses, registered nurses, nurses with academic degrees), no significant differences were found in the prevalence of violence toward them. However, a significant difference (*P* = 0.0001) was found among different types of physicians; the more senior the physician, the less he/she is exposed to violence. Indeed, none of the interns escaped from being exposed to violence by patients or companions, as seen in Table 5.

**Table 5:** Physician’s academic rank

	N	Patient	Companion
Senior physician	127	54 (42%)	68 (53%)
Resident physician	82	58 (71%)	66 (80%)
Intern	21	20 (95%)	18 (86%)
Total	230	132 (57%)	152 (66%)
*P* value		*P* = 0.0001	*P* = 0.00004

Using a t-test, an association was found between professional seniority and exposure to violence categorized as passive aggressive patient behavior, equipment destruction by companion, and sexual harassment by both patients and companions (Table 6).

**Table 6:** Years of seniority and association with exposure to violence, total population

Type of violence	By whom	Prevalence		Seniority (years) average + SD		Median		*P* value
		Yes	No	Yes	No	Yes	No	
Verbal violence	Companion	311	341	13.3 ± 10.6	14.95 + 11.7	10	12	*P* = 0.056
Passive aggressive	Patient	317	334	13.1 ± 11	15.2 ± 11	10	13	*P* = 0.019
Equipment destruction	Companion	57	592	10.3 ± 9.4	14.5 ± 11.2	7	12	*P* = 0.013
Sexual harassment	Patient	44	608	8.6 ± 8.6	14.6 ± 11.2	5.75	12	*P* = 0.001
	Companion	32	614	8.6 ± 8.5	14.5 + 11.2	5.25	12	*P* = 0.003

These results were statistically significant. Namely, the less seniority one has, the more he/she is exposed to the types of violence mentioned. The rate of verbal violence demonstrated by companions of patients was high. The association between professional seniority and this type of violence, however, had borderline significance (*P* = 0.056). As can be seen in Table 7, this association is found among both physicians and nurses.

**Table 7:** Years of seniority and association with exposure to violence, nurse vs physician

Type of violence	By whom	Victim	Prevalence		Seniority (years) average ± SD		Median		*P* value
			Yes	No	Yes	No	Yes	No	
Verbal violence	Companion	Nurse	224	208	14.5 ± 10.9	14.5 ± 11.9	12	11	0.980
		Physician	84	131	9.89 ± 9.2	15.1 ± 11.1	5	15	0.001
Passive aggressive	Patient	Nurse	223	209	14 ± 11.1	15.1 ± 11.6	10	12	0.291
		Physician	92	122	10.9 ± 10.5	15.3 ± 10.7	6	15	0.003
Sexual harassment	Patient	Nurse	35	397	9 ± 8.8	15 ± 11.5	6	12	0.02*
	Companion		25	401	9.5 ± 9	15 ± 11.5	6	12	0.018*
Sexual harassment	Patient	Physician	8	207	4.6 ± 3.5	13.7 ± 10.8	5.3	11	0.02*
	Companion		6	209	3.3 ± 2	13.6 ± 10.8	3	10	0.013*

*Mann–Whitney Test

### Correlation between exposure to violence and hospital departments

A distribution of respondents by departments reveals that over 50% of respondents, in all departments, reported having been exposed to violence in the past 6 months. A significant difference was found between the rates of exposure to violent incidents in the department of emergency medicine, ambulatory care and clinics on one hand compared to the departments of internal medicine, surgery, oncology and intensive care on the other hand. For example, the risk that a nurse in the emergency room would be exposed to violence was 5.5 times greater than that of a nurse in the department of internal medicine. No significant difference was found between the ambulatory care and clinics and the emergency room in the risk of exposure to violence (Table 8).

**Table 8:** Prevalence of exposure to violence by fields of specialty

Fields of specialty	Respondents	Violence perpetrated by patient	Violence perpetrated by companion
Emergency room	Physician	3 (75%)	2 (50%)
	Nurse	23 (92%)	23 (92%)
Out clinics	Physician	1 (100%)	1 (100%)
	Nurse	7 (84%)	33 (72%)
Day care units	Physician	15 (68%)	14 (64%)
	Nurse	26 (72%)	27 (75%)
Surgery departments	Physician	46 (63%)	49 (67%)
	Nurse	82 (68%)	88 (73%)
Internal departments	Physician	48 (53%)	61 (67%)
	Nurse	58 (57%)	68 (67%)
Intensive care units	Physician	8 (44%)	9 (50%)
	Nurse	33 (60%)	42 (76%)
Oncology departments	Physician	11 (50%)	16 (72%)
	Nurse	33 (54%)	30 (50%)

Nurses working in the ER were more exposed to violence than other nurses (Pearson Chi-Square 20.756^b^, df-6, *p* = 0.002), while location of work was not found to affect exposure to violence for physicians.

### Background variables of staff members and exposure to violence - multivariate regression

In order to examine the independent associations between any exposure to violence by a patient or companion in the previous 6 months and the background characteristics of physicians and nurses, a multivariate analysis was performed. As seen in Table 9, the analysis found no significant differences between men and women, but the age of the professional was found to be significant. Namely, older healthcare personnel had a lower risk of being exposed to violence. For every added year, the risk of being exposed to violence was 4% lower than the previous year.

**Table 9:** Multivariate regression model - the relationship between exposure to violence and background variable of staff members

Variable	Violence perpetrated by companion				Violence perpetrated by patient			
	95% C.I. for OR		Adjusted OR	Sig.	95% C.I. for OR		Adjusted OR	Sig.
	Upper	Lower			Upper	Lower		
Gender^1^	1.24	.47	.76	.28	1.60	.64	1.01	.94
Age	.99	.93	.96	.04	.99	.92	.95	.01
Position^2^				.00				.03
Medical director	8.12	.47	1.96	.35	2.39	.23	.74	.61
Senior physician	.709	.20	.38	.00	.82	.24	.44	.01
In-house physician	2.95	.72	1.46	.29	2.99	.83	1.58	.15
Junior physician	17.42	.21	1.93	.55	.	.00	9200	.99
Head nurse	9.75	.95	3.05	.06	5.05	.65	1.82	.24
Head nurse deputy	1.57	.30	.68	.37	2.19	.42	.96	.93
Professional Seniority	1.03	.95	.99	.80	1.05	.97	1.01	.49
Departmental Seniority	1.06	.99	1.03	.10	1.04	.97	1.01	.53
Departmental procedure^3^				.24				.26
There is a procedure	2.39	.80	1.38	.24	1.88	.65	1.11	.69
No procedure	2.16	.90	1.40	.12	1.14	.49	.75	.18
Course participation	1.79	.52	.96	.92	1.66	.51	.95	.79
Division^4^				.01				.00
Internal	.85	.04	.18	.03	.67	.05	.18	.01
Surgery	.93	.04	.20	.04	.87	.06	.24	.03
Oncology	.46	.02	.09	.00	.68	.04	.18	.01
Intensive care	1.10	.04	.22	.06	.81	.05	.21	.02
Clinics	1.29	.04	.24	.09	3.65	.17	.80	.77
Day care	1.70	.06	.34	.19	2.29	.13	.56	.42

Reference Categories: ^1^Female ^2^Staff nurse ^3^Do not know ^4^ER

Profession was also an independent risk factor. The risk of a physician being exposed to violence is less than half that of a nurse. A senior physician has a much lower risk of being exposed to violence than a nurse.

None of the demographic and professional variables (age, being a senior physician, and working in internal medicine, surgery and oncology) were found to increase the risk of exposure to violence by patients, except in intensive care units.

The other variables, such as having departmental procedures and regulations on coping with violence or participating in a violence prevention workshop, were not found to be risk factors.

## Discussion

This study has gone into the basket of types of violence and measured the frequency of the types that occur in different departments in a major general hospital in Israel. Within this basket different types of violence are directed towards different types of staff by patients and companions. Thus, this study extends pervious knowledge of the phenomenon in a significant way.

Previous studies found high levels of violence in Israeli ERs. This study is the first in Israel to examine the extent of violence perpetrated towards nurses and physicians in a general hospital, not only in ERs, and the study found that violence is a common occurrence in all of the departments studied. The rates of exposure to violence found in EDs in our study was similar to the rates of violence reported by Derazon et al., (1999) mentioned above, who found a high proportion of violence in the ED in a single Israeli hospital (74%). Our findings are also consistent with those of Landau and Bendalak (2010), who found that nurses experience the most violence, most of which is verbal and about 10% physical.

With regard to ***the types of violence,*** physicians and nurses are exposed to various types of violence in the hospital’s various departments, as seen, for example in Table 4, nurses experience higher levels of explicit threats and verbal violence, and, in Table 7, higher levels of sexual harassment. In all departments, a remarkably high rate of exposure to all types of violence in the previous 6 months was reported, ranging from 52%- 96%. Approximately 700 incidents of passive aggressive behavior, 680 incidents of verbal violence and 81 incidents of sexual harassment took place during the study period. We also found that for verbal violence, explicit threats and passive aggressive behavior between individual staff experience three or more occurrences at rates varying between 5 and 20% (Table 3).

Passive aggressive behavior, heavily represented in our study, is often not included in studies of violence. It could be argued that this inflates the prevalence of violent events in our study. However, in a companion study [24], this type of behavior was not only frequently mentioned in our focus groups, but also seen to contribute to the unfolding of violent events. Perhaps the tendency to discount passive aggressive behavior should be re-visited, and its role in violent episodes needs to be further studied.

It should be emphasized that although sexual harassment is the least reported, it is a worrisome and troubling phenomenon. In such cases, nurses might experience a double threat: gender and professional.

***With regard to nurses and physician’ exposure to violence,*** as shown in the results, there is a difference between types of behavior directed towards nurses as opposed to physicians. This difference may be rooted in their gender and professional prestige. Perhaps patients and their companions allow themselves to behave more blatantly towards nurses than to physicians. Furthermore, passive aggressive behaviors require longer interactions to manifest themselves, and these are present in nurse-patient interactions as opposed to shorter patient- physician encounters. This exposure to violence seems to affect nurses’ daily practice, has an impact on stress and productivity, and also increases their intention to leave their job [25, 26].

Our findings indicated that nurses are more exposed to verbal violence and to passive aggressive behavior than physicians as indicated in other studies as well [27, 28]. This might be explained by the fact that the nurses are on the front line, working 24/7 at the patient’s bedside.

Both nurses and physicians were more exposed to violence from companions than from patients. Companions may be acting out of a sense of responsibility or burden to protect the patient. These behaviors should be acknowledged and addressed by the staff and health system leaders to prevent miscommunication and harmful events.

When comparing among the **nurses** themselves (registered nurses with academic degrees, other registered nurses, and licensed practical nurses), no differences were found in the prevalence of violent incidents. This is probably due to the fact that in practice, all nurses work by the patient’s bedside, notwithstanding their academic certification. Patients and those accompanying them do not discern the hierarchy among nurses. This is different from the findings of previous studies, which showed that although both RNs and LPNs are frequently subjected to physical and non-physical forms of violence, LPNs are more exposed and at greater risk [29, 30]. The result here may be related to the fact that our sample did not include enough licensed practical nurses.

As for ***professional seniority*** among **physicians**, only a few studies have been published by physicians or refer to a population of physicians, perhaps due to the lower prevalence of violence as shown in our study. The current study displays a significant association between professional seniority of physicians and certain types of violence. Namely, the more senior the physician the less he/she is exposed to passive aggressive behavior and sexual harassment. Notably, the current findings show that more companions than patients demonstrate verbal violence and passive aggressive behavior towards physicians.

Similarly, an epidemiological study conducted in Japan found that 24% of physicians reported having been subjected to verbal violence and 5% reported physical violence. A significant association was found between age and verbal violence and between gender and physical violence. Physicians younger than 30 experienced more verbal violence and female physicians experienced more physical violence [31]. Whether relative youth and being female engender more perceptions of vulnerability, on the part of perpetrators and victims, calls for further study.

***Regarding hospital departments*** it must be emphasized that a significant difference was found between the extents of exposure to violence in the emergency room versus other departments. It is known that emergency staff is more subject to violence at rates sometimes exceeding 90% for verbal violence [32]. Notably, in our study no significant difference was found between the outpatient clinics and the emergency room, suggesting the possibility that relatively long waiting times, that characterize both of these settings may be a catalyst for violence. The major difference between the emergency room and the outpatient clinic is the urgency of care required, but in both, a lengthy waiting time is usually required. This waiting time component might have triggered violent behavior, unrelated to one’s urgent clinical condition.

Other reasons for violent episodes in the emergency room’s are mentioned in the literature such as, crowding/workload, shortages of both nursing and medical staff, care of patients with dementia and emergency room procedures [32].

Another conspicuous, albeit unsurprising finding is the low exposure to violence in the oncology departments. This finding might be linked to the nature of the relationship between nurse/physician and patients in the case of progressive life-threatening illnesses that require lengthy treatments. Another explanation for the low rate of violence in oncology departments is the comfortable physical conditions provided to the patients and families (private rooms with a separate air-conditioning system, television etc.). These physical conditions are known as violence inhibitors [33].

### Implications

This study was performed as a case study in a single tertiary hospital, although it has implications for other Israeli hospitals and cross nationally as well.

Violence has been treated too often as a monolithic concept. The variety of violent behaviors perpetrated by different actors and their frequencies in different departments that were uncovered by this paper must be taken into account in formulating policy interventions.

First and foremost, implementing interventions to increase the safety of the healthcare environment for both patients and employees should cover the entire healthcare system both in term of departments and personnel. True, nurses, especially in the ER, are at the front and it may necessitate aiming more training programs to prevent and manage crises in the hospital settings. But beyond this we have revealed that such interventions should prioritize young nurses. Yet, these programs as well as other safety measures which are designed to create a safe environment should be implemented and taught to all heath care workers. So when the American Nurse Association declared recently, “*a safe and healthy inter professional work environment should be created and sustained for RNs and all health care professionals*” [34]. *The evidence from our research can refine this in terms of targeting the efforts.*

There is a lack of research evidence for violence prevention program effectiveness [1]. Perhaps this is due to failure to recognize and prioritize those departments, actors and victims that are most likely to become involved in violence. As mentioned above, only 19.2% of the staff in the hospital studied here reported that their department had written guidelines for handling violence. In a companion study [24], we found that most of the existing material relating to violence in the hospital related to calling for intervention of security personnel, indicating that few tools have been developed for prevention and coping. This lack may be due, in turn, to a lack of in depth understanding of the essence of hospital violence, its causes and the forms it takes.

Future research should focus on refining content and methods of teaching all intervention programs to take account of the multi-dimensional nature of hospital violence revealed in this paper. No doubt, there is a need for a carefully planned program that would incorporate a multifaceted, multidisciplinary approach to violence reduction. Such a comprehensive approach should include unified guidelines for violence prevention, promotion of legislation, managerial support, and an effective reporting system. These measures which would include staff across all levels, continuous training programs as well as installation of security cameras, metal detectors, etc., should be instituted, [1, 35]. In addition, guidelines need to relate to micro contingencies. For example, in emergency room and ambulatory clinic settings, perhaps most important is to calm anxiety and tension that come from waiting times, in particular by reducing uncertainty regarding time to treatment. But reducing such uncertainty may be more difficult in the emergency room, where waiting times exacerbate the more panicked and shock filled atmosphere surrounding emergency care. Thus, the documentation provide by this study of the different patterns of violence occurring in different departments, even when frequency is similar across two departments, can shape different interventions responding to different contingencies.

Perhaps, it might be possible to learn from the success of the oncology staff who has reported low exposure to violence. Thus, in an attempt to further develop and enhance the effectiveness of the training programs, each department’s unique characteristics could be addressed based on the added value of this study as a learning tool to achieve preventive measures.

The authors of this article believe that management commitment is essential to enhance these recommendations. Managers need to understand that a change in policy can be implemented only when the manager becomes a service model, in which he or she serves the patients and companions and the hospitals’ employees.

Moreover, violence as a social phenomenon encountered by hospital workers should be addressed at all operational and organizational levels of the hospital.

Therefore, hospital management must clearly inform the workers and patients about the policy regarding violence, best practices and rules of behavior in the organization.

Any violent event must enter a transparent reporting system as well as providing a progressive framework for lesson learning. In addition, violence prevention and management programs should be incorporated into standard organizational procedures such as worker evaluations, service promotion projects, etc.

### Limitations

This study was performed in a single medical center; similar studies should be carried out in additional Israeli hospitals. They can build upon the concepts and measures developed as part of this study. The current study included only nurses and physicians. As violence in the health systems and its causes, involve the entire organization, the role and perceptions of other actors, such as administrators and security personnel, (not to mention patients and their companions), needs to be addressed. We do this is a companion study [24].

The current study focused on extent of exposure per staff member. First, since all types of violence were weighted equally in our summary measure of exposure, the severity of violence is not brought to bear and this might skew some of our comparisons regarding levels of exposure among different groups, such as physicians and nurses. Second, as the number of patients to whom a staff member is exposed varies by department and profession, the data presented here do not provide an indication of the rate of violence per patient. That could be the subject of a complementary study.

## Conclusion

This study has shown that violence occurs in all hospital departments and that there is a difference in the degree of exposure to violence between physicians and nurses and between departments. The definition of the various types of violence is important to create a uniform discourse both at the research and at the organizational levels. Although this study was performed in a single large hospital, it provides important insights into a worrisome phenomenon both nationally and globally. An executive commitment is necessary and critical for a thorough organizational change to occur. The findings could enable policy makers in the Israeli healthcare system to formulate and implement interventions on a national level and to promote leaders’ commitment for violence prevention and management.

## Abbreviations

ER: Emergency room
LPN: Licensed practical nurses
RN: Registered nurses
